# Preparation of Amphiphilic Chitosan-Loaded Bentonite Adsorbent and Its Performance in Removing Organic Matter from Coking Wastewater

**DOI:** 10.3390/polym15061588

**Published:** 2023-03-22

**Authors:** Zhou Zhu, Haiqun Kou, Yuchang Zhou, Xindian Lan, Meiying Yu, Haonan Chen

**Affiliations:** 1 School of Ecology and Environment, Yuzhang Normal University, Nanchang 330103, China; 2 Key Laboratory of Green New Materials and Industrial Wastewater Treatment of Nanchang City, Yuzhang Normal University, Nanchang 330103, China

**Keywords:** chitosan, bentonite, coking wastewater, adsorbent, hydrophobic modification

## Abstract

An amphiphilic chitosan-loaded bentonite adsorbent (C18CTS−BT) was prepared for the efficient removal of organic matter from coking wastewater. The structure and surface morphology of adsorbents were characterized by FT−IR, XRD, and SEM. The removal of those organics by C18CTS−BT was investigated by comparing the adsorption performances of C18CTS−BT with bentonite (BT) and chitosan-loaded bentonite (CTS−BT). The results showed that compared with BT and CTS−BT, C18CTS−BT showed the performance advantages of having a low dosage, wide pH range, and short adsorption equilibrium time. The optimized treatment process was as follows: the adsorbent dosage was 1.5 g·L^−1^, the adsorption time was 60 min, and the pH of the system was 7.0. The chemical oxygen demand (COD) of the coking wastewater treated with BT, CTS−BT, and C18CTS−BT decreased from 342 mg·L^−1^ in the raw water to 264 mg·L^−1^, 218 mg·L^−1^, and 146 mg·L^−1^, corresponding to COD removal rates of 22.81%, 36.26%, and 57.31%, respectively. The results of GC−MS analysis also confirmed that C18CTS−BT could remove most of the organic compounds in coking wastewater, especially long−chain alkanes and their derivatives. The hydrophobic modification of the adsorbent material can effectively improve the removal performance of organic compounds from coking wastewater.

## 1. Introduction

Coking wastewater is problematic industrial organic wastewater produced in the coking industry during the production of coke from bituminous coal [1]. Due to the poor biochemical properties of coking wastewater, the chemical oxygen demand (COD) of effluent after biochemical treatment cannot reach the discharge standards; thus, it must be further treated in depth [2,3]. The deep treatment technologies for coking wastewater mainly include advanced oxidation [4], coagulation [5], adsorption [6], membrane treatment [7], etc. Among the other methods, the adsorption method has the advantages of it being a simple process, recyclable, having a fast adsorption rate, and producing no secondary pollution, etc., and has become a hot research spot in this field. Therefore, it is of great economic and social benefit to develop highly efficient, inexpensive, and green adsorbents to achieve the water quality characteristics of coking wastewater.

In recent years, some natural adsorbent materials have been increasingly favored by researchers, such as bentonite [8], lignin [9], chitosan [10], and biomass carbon [11]. Among the other types, both bentonite (BT) and chitosan (CTS) are widely studied environmentally friendly materials. BT is a natural, colloidal, and hydrated aluminosilicate that occurs as a clay sediment with significant amounts of minerals from the smectite group [12]. BT has a large specific surface area, which is beneficial in improving the adsorption capacity and efficiency when it is being used as a wastewater adsorbent [13]. However, although BT is rich in mesopores and has a large specific surface area, its adsorption performance is easily affected by environmental factors [14]. For coking wastewater, the high fluctuation in wastewater quality has limited its application in the field of water treatment. CTS is a polysaccharide and the second most abundant natural polymer [15]. The advantages of chitosan include the following characteristics: biodegradability, low toxicity, and easy chemical modification [16]. CTS has been used in many fields such as food, medicine, cosmetics, and wastewater treatment [17]. Although the specific surface area of CTS is relatively small, its molecular structure is rich in active groups, which can achieve the migration of pollutants from wastewater through the interaction between acting groups on the adsorbent surface and pollutants [18]. Researchers have developed CTS−loaded BT adsorbent by combining the advantages of BT and CTS, and the studies show that the treatment effect of CTS−loaded BT adsorbent is better than that of single BT or CTS [19,20,21]. These findings have laid the foundation for the application of CTS−loaded BT adsorbent in coking wastewater. On this basis, how to further improve the adsorption performance of these adsorbents has become the focus of research in this field.

In this paper, hydrophobically modified O−octadecyl−CTS (C18CTS) was prepared by introducing long−chain alkanes with a strong hydrophobic effect into the molecular structure. Then, an amphiphilic adsorbent (C18CTS−BT) was prepared by loading C18CTS onto BT with microwave assistance. By comparing the performances of different adsorbents for COD removal from coking wastewater, the effect of hydrophobic modification on their adsorption performance was investigated to provide theoretical guidance for the application of natural adsorbent materials in coking wastewater.

## 2. Materials and Methods

### 2.1. Materials

Chitosan (CTS, deacetylation degree 85%), sulfuric acid (H_2_SO_4_, mass fraction 98%), and bromo−octadecane (97% purity) were purchased from Heowns Biochemical Technology Co., Ltd. (Tianjin, China). Benzaldehyde, glacial acetic acid, sodium hydroxide (NaOH), anhydrous ethanol, isopropanol, acetone, and bentonite (BT) were purchased as analytical pure reagents from Sinopharm Chemical Reagent Co., Ltd. (Shanghai, China). The coking wastewater was taken from the secondary sedimentation tank effluent of a coking plant in Jiangxi Province after biochemical treatment. After testing the water sample in our laboratory, the water quality data were obtained as follows: the pH value was 7.2, that of COD was 342 mg·L^−1^, that of ammonia nitrogen was 8.82 mg·L^−1^, that of total nitrogen was 100.6 mg·L^−1^, and that of total phosphorus was 1.73 mg·L^−1^.

### 2.2. Preparation of C18CTS−BT

**Figure 1:**
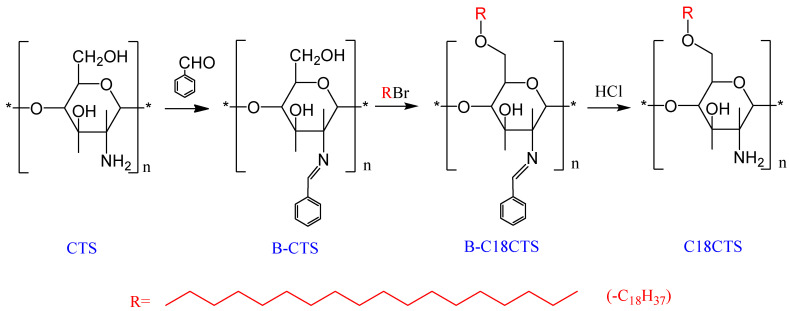
Preparation route of C18CTS.

Amino-protected benzaldehyde chitosan Schiff base (B−CTS) was first prepared according to the literature [22]. Following this, 2.00 g of B−CTS, 4.00 g of potassium hydroxide, and 24 mL of isopropanol were placed in a 100 mL three-necked flask, and the water bath was set at 40 °C, and then thermostated for 1 h to alkalize B−CTS. After the temperature was raised to 60 °C, 6 mL of bromooctadecane was added to the flask with continuous stirring, and the reaction was kept at a constant temperature for 8 h. After the reaction, the product was filtered and washed repeatedly with anhydrous ethanol and ultra-pure water. Hydrophobically modified O−octadecyl−benzaldehyde chitosan (B−C18CTS) was obtained by drying it to a constant weight. After soaking and stirring B−C18CTS in dilute hydrochloric acid for 2 h, the product was extracted with anhydrous ethanol as the solvent for 12 h using a Soxhlet extractor to finally obtain O−octadecyl−chitosan (C18CTS). The preparation route of C18CTS−BT is shown in Figure 1.

Following this, 1.00 g of C18CTS was added to 100 mL of glacial acetic acid solution (1%, *v*/*v*) and stirred thoroughly to dissolve it completely. Then, 25.00 g of BT was added to the solution and stirred at 45 °C for 1 h to form a paste. The mixture was placed in a microwave reactor (FCMCR−3S, KeRui Instrument Co., Gongyi, China) set at 300 W with a heating time of 10 min. The final adsorbent C18CTS−BT was obtained by drying, grinding, and sieving (100 mesh), respectively. A chitosan−loaded bentonite adsorbent (CTS−BT) was also prepared using the same method in comparative studies.

### 2.3. Characterization

The characteristic functional groups of the adsorbent were analyzed by Fourier Transform Infrared (FT−IR). FT−IR was performed by using Nicolet 6700 infrared spectroscopy (Thermo, Waltham, MA, USA). An X−ray diffractogram (XRD) of the sample was obtained by means of an Ultimate IV X−ray diffractometer (Rigaku, Japan), which was operated at 40 kV and 40 mA of CuKα radiation (λ = 0.154 nm). The surface morphology of the adsorbent was captured by scanning electron microscopy (SEM). SEM images were obtained on a JSM−7900F electron microscope instrument (JEOL, Tokyo, Japan). The pore property of the adsorbent was characterized by a fully automated specific surface and porosity analyzer (Nova 4000e, Quantachrome, Boynton Beach, FL, USA). The surface area and pore diameter of the adsorbents were obtained by Brunner−Emmet−Teller (BET) and Barret–Joyner−Halenda (BJH) methods [23]. Gas chromatography–mass spectrometry (GC−MS, 7890A-5975C, Agilent, Santa Clara, CA, USA) was used to analyze the organic composition of the coking wastewater before and after treatment. Before the GC−MS test, dichloromethane was used as the extractant to extract organic matter in the coking wastewater, and then the extract was concentrated by nitrogen purging.

### 2.4. Determination of COD Removal Rate of Wastewater

A total of 100 mL of coking wastewater was poured into a beaker, and then the pH value of the wastewater was adjusted with a mass fraction of 10% H_2_SO_4_ or 2 mol·L^−1^ NaOH solution. A certain amount of adsorbent was added to the wastewater and shaken in a constant temperature shaker (THZ−312, YiNeng Experimental Instrument Factory, Changzhou, China) at 25 °C and 200 rpm for a certain time. The wastewater was filtered with a 0.45 μm micro−porous membrane, and the filtrate was taken for measurement. The COD of coking wastewater before and after treatment was measured using a multi−parameter water quality analyzer (LH-3BA, Beijing Lianhua YongXing Science and Technology Development Co., Ltd., Beijing, China), and the COD removal rate of coking wastewater was used to evaluate the adsorption performance of the adsorbent. The COD of each group of wastewater was measured three times, and then the average COD was obtained. The COD removal rate was calculated according to Equation (1).

(1) $$COD\;removal\;rate=\frac{COD_{0}-COD_{m}}{COD_{0}}\times 100\%$$

where *COD*_0_ and *COD_m_* were the *COD* of coking wastewater before and after treatment, mg·L^−1^, respectively.

## 3. Results and Discussion

### 3.1. FT-IR Analysis

**Figure 2:**
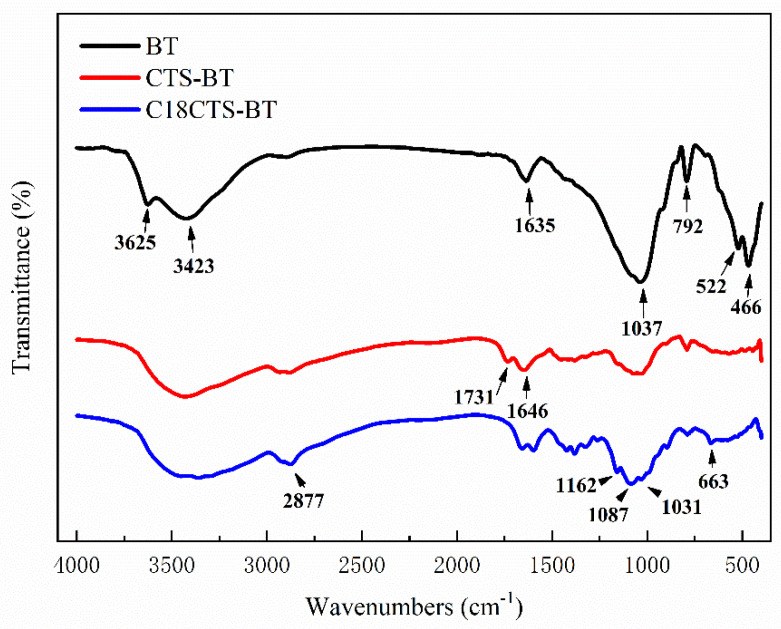
FT−IR of the adsorbents.

Figure 2 shows the FT−IR of three different adsorbents, BT, CTS−BT, and C18CTS−BT. For BT, the absorption peak at 3625 cm^−1^ is the absorption peak of the stretching vibration of Al−O−H. The absorption peaks at 3423 cm^−1^ and 1635 cm^−1^ are attributed to stretching vibration and bending vibration of water molecules O−H between the crystal layers, respectively. The absorption peaks at 1037 cm^−1^ and 792 cm^−1^ are Si−O−Si antisymmetric stretching vibration absorption peaks in BT. The absorption peaks at 522 cm^−1^ and 466 cm^−1^ are associated with the coupling vibrations of Si−O−M (metal ions) and M−O. For CTS−BT, broader absorption peaks from 3260 cm^−1^ to 3500 cm^−1^ are the overlapping absorption peaks of stretching vibrations of −NH_2_ and −OH. The C=O stretching vibration and N−H bending vibration absorption peaks at 1731 cm^−1^ and 1646 cm^−1^, respectively, are characteristic absorption peaks of the amide group. These are caused by the degree of incomplete deacetylation of CTS, indicating the successful preparation of CTS−BT. For C18CTS−BT, its IR spectrum is similar to that of CTS−BT. The difference is that the absorption peak of the stretching vibration of C−H at 2877 cm^−1^ is significantly enhanced. The characteristic absorption peaks at 1031 cm^−1^, 1087 cm^−1^, and 1162 cm^−1^ are attributed to the bending vibration of −OH and the stretching vibration of C−O−C. In addition, the characteristic absorption peak at 663 cm^−1^ is attributed to the presence of long-chain alkyl groups. The above characteristic peaks indicate that C18CTS−BT was prepared successfully.

### 3.2. XRD Analysis

**Figure 3:**
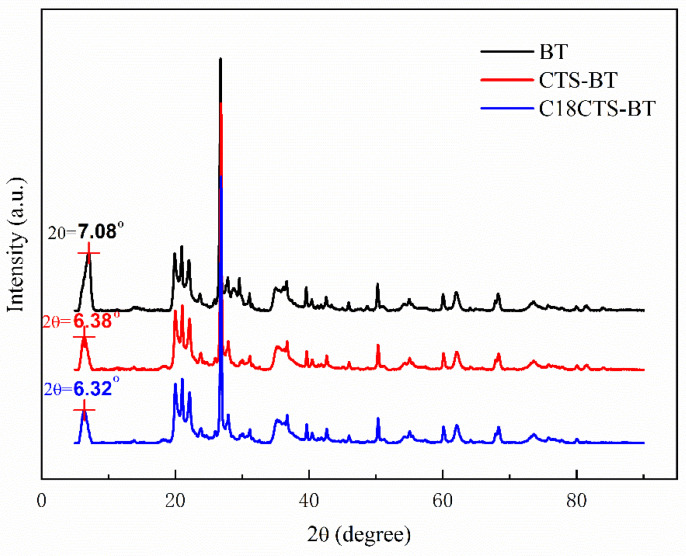
XRD patterns of the adsorbents.

In order to further analyze the physical phase characteristics of the adsorbents, XRD tests were performed on three different adsorbents. From Figure 3, it can be seen that the XRD patterns of the three different adsorbents correspond to different diffraction peaks at different diffraction angles, and the diffraction angle corresponding to the first peak is generally used to calculate the crystal layer spacing. For BT, CTS−BT, and C18CTS−BT, the diffraction angles corresponding to the first peak in their diffraction patterns are 7.08°, 6.38°, and 6.32°, respectively. Based on Bragg’s law [24], the crystal layer spacings of BT, CS−BT, and C18CS−BT are calculated to be 1.2485 nm, 1.3853 nm, and 1.3985 nm, respectively. The results show that the crystal layer spacing of CTS−BT and C18CTS−BT is significantly larger than that of BT, indicating that CTS or C18CTS is effectively intercalated into the crystal layer of BT to expand its crystal layer spacing. However, the crystal layer spacings of CTS−BT and C18CTS−BT are approximately comparable, which indicates that the hydrophobically modified CTS had almost no effect on crystal layer spacing. It can also be seen from Figure 3 that the XRD patterns of the three different adsorbents approximately correspond to the same diffraction peaks at different diffraction angles, showing that the modification had no effect on the basic structure of BT, and only the crystal layer spacing changed.

### 3.3. SEM Analysis

**Figure 4:**
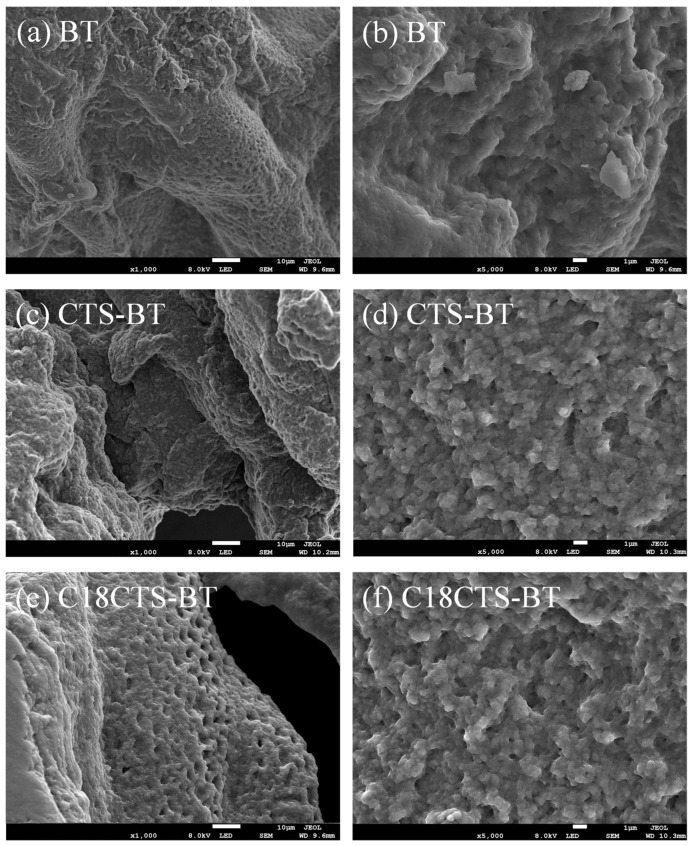
SEM photographs of the adsorbents.

The surface morphologies of the three different adsorbents were characterized by SEM, as shown in Figure 4. It can be seen from Figure 4a,b that the surface of BT has some “folds” and pore channels, and thus it has some adsorption capacity. Figure 4c,d shows that the surface of CTS−BT is significantly rougher and the number of pores is significantly higher. From Figure 4e,f, it can be seen that the surface of C18CTS−BT also shows a similar uneven and fluffy structure.

### 3.4. Pore Property Analysis

**Figure 5:**
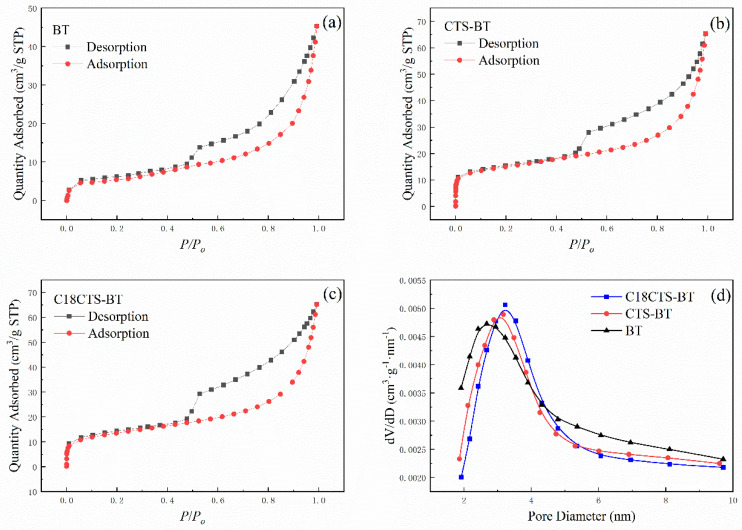
N_2_ adsorption–desorption isothermal curve and pore size distribution curve of the adsorbents ((**a**–**c**) show the N_2_ adsorption–desorption isothermal curves for BT, CS–BT and C18CS–BT, respectively; (**d**) shows the pore size distribution curves for three different adsorbents).

The N_2_ adsorption–desorption measurements of BT, CTS–BT, and C18CTS–BT are shown in Figure 5. It can be seen from Figure 5a–c that these adsorbents contain a certain number of mesopores since their N_2_ adsorption–desorption isotherms show a hysteresis loop [25]. According to hysteresis loop classification proposed by the International Union of Pure and Applied Chemistry (IUPAC), the hysteresis loop types of BT, CTS–BT, and C18CTS–BT belong to the H3 type [26]. Based on the Brunner–Emmet–Teller (BET) method, the specific surface areas of BT, CTS–BT, and C18CTS–BT are determined to be 34.63 m²·g^−1^, 49.87 m²·g^−1^, and 51.93 m²·g^−1^, respectively. As can be seen in Figure 5d, the pore size distribution curves of CTS–BT and C18CTS–BT are significantly shifted to the right relative to BT, which indicates that the pore sizes of CTS–BT and C18CTS–BT became larger. In addition, the differences between C18CS–BT and CS–BT in terms of specific surface area and pore size distribution are not significant, indicating that the hydrophobic modification has a little effect on the pore structure of the adsorbent.

### 3.5. Effect Factors of COD Removal Rate

#### 3.5.1. Effect of Adsorbent Dosage

**Figure 6:**
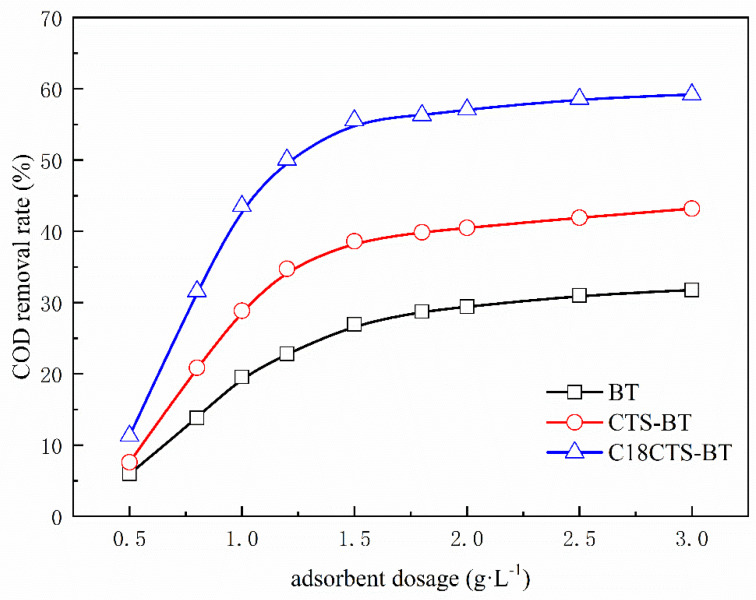
Effect of adsorbent dosage on removal rate of COD (pH = 7.0; adsorption time is 2 h).

The dosage of adsorbent is one of the important factors affecting its adsorption performance, and also, it is an important reference for the operating cost of the adsorbent actually applied in wastewater treatment [27]. It can be seen from Figure 6, with the increase in adsorbent dosage, the COD removal rate of coking wastewater by all three adsorbents shows a characteristic of first increasing, and then stabilizing. As the dosage of the adsorbents increases, their adsorption sites increase, leading to an increase in the rate of COD removal. Under the premise that the concentration of organic matter in coking wastewater is certain, the concentration of organic matter in the wastewater gradually decreases with the increase of adsorbent dosage, which stabilizes the removal rate of COD from coking wastewater by the adsorbents. The COD removal rate of both CTS–BT and C18CTS–BT is higher than that of BT with the same adsorbent addition, indicating that the adsorption performance of the modified adsorbent on the organic matter is significantly improved. Further comparison reveals that the adsorption performance of C18CTS–BT is further improved after hydrophobic modification. When the dosage of C18CTS–BT is increased from 1.5 g·L^−1^ to 3.0 g·L^−1^, the COD removal rate increases from 55.56% to 59.21%, and the increase is no longer significant. Considering the application cost and the convenience of the comparative study, the dosage of all three adsorbents is determined to be 1.5 g·L^−1^.

#### 3.5.2. Effect of pH Value

**Figure 7:**
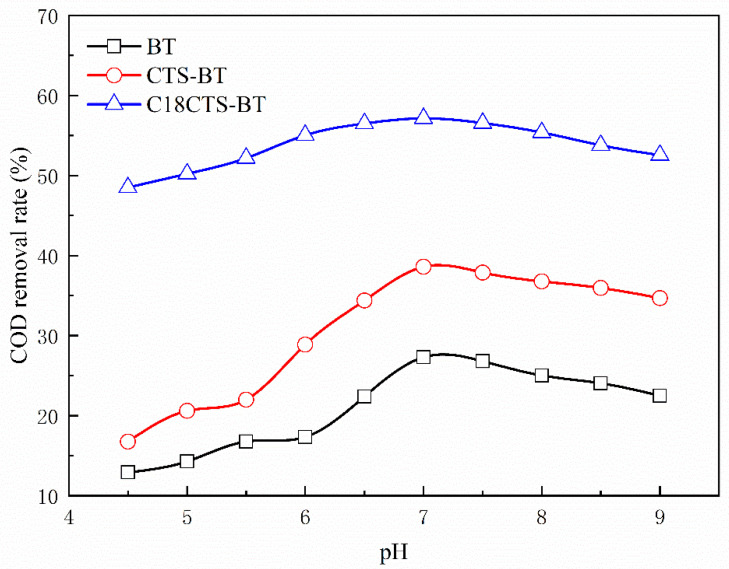
Effect of pH value on removal rate of COD (adsorbent dosage is 1.5 g·L^−1^; adsorption time is 2 h).

Figure 7 shows the effect on the COD removal rate of the three adsorbents when the pH value of the wastewater varied in the range from 4.5 to 9.0. When the pH value of the system is 4.5, the lowest COD removal percentages are achieved for BT, CTS–BT, and C18CTS–BT: 12.72%, 16.45%, and 48.34%, respectively. The COD removal rates of all the adsorbents show an increasing trend as the pH of the system increased. When the system’s pH was 7.0, the highest COD removal rates of 27.48%, 38.55%, and 57.43% were obtained for BT, CTS–BT, and C18CTS–BT, respectively. When the pH of the system was higher than 7.0, the removal rates of all the adsorbents show a decreasing trend. When the system’s pH was 9.0, the COD removal rates of BT, CTS–BT, and C18CTS–BT were 22.51%, 34.68%, and 52.61%, respectively. Under acidic conditions, H^+^ in the solution occupies the adsorption sites of the adsorbent through electrostatic interactions, and this “competitive adsorption” leads to a decrease in the removal of COD from coking wastewater by the adsorbent [28]. In alkaline conditions, the form of organic matter present in coking wastewater changes, such as the presence of some hard–to–biodegrade polyphenols in the form of anions. However, the surface of the bentonite-based adsorbent is negatively charged and the electrostatic repulsion leads to a decrease in its removal of organic matter.

Further analysis reveals that the COD removal rate of C18CTS–BT fluctuates from 48.34% to 57.43% when the pH value of the system is in the range of 4.5~9.0. The above results indicate that the pH of the system has a less significant effect on the COD removal rate of C18CTS–BT compared with those of the other adsorbents, indicating that it has a broader pH application range. Given the introduction of strong hydrophobic groups, the surface of C18CTS–BT in the system formed water–repellent micro–regions, which weaken the “competitive adsorption” of the strongly hydrated H^+^ in the acidic system. In alkaline conditions, the COD removal rate of C18CTS–BT is reduced due to a change in the form of adsorbent present in the wastewater. In addition, the COD removal rate of C18CS–BT is higher than that of the other adsorbents at different pH values. Taking this into consideration, the pH value of the system is determined to be 7.0.

#### 3.5.3. Effect of Adsorption Time

**Figure 8:**
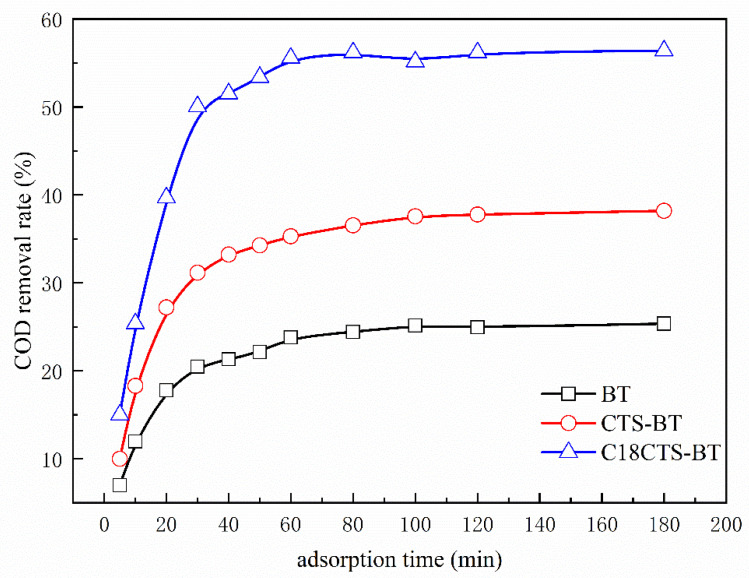
Effect of adsorption time on removal rate of COD (adsorbent dosage is 1.5 g·L^−1^; pH = 7.0).

The adsorption time is one of the important process parameters for using the adsorbent for practical applications. Figure 8 shows the curves of COD removal rate versus adsorption time for coking wastewater with three different adsorbents. It can be seen from Figure 8 that with the increase in adsorption time, the COD removal rates of the three adsorbents show three distinct phases: (1) a sharp increase phase; (2) a slow increase phase; (3) a stabilization phase. This is because there are a large number of adsorption sites on the surface of the adsorbent at the initial stage, and the organic matter in the coking wastewater diffuses to the surface of the adsorbent through the concentration difference, resulting in a relatively large adsorption rate at this stage. With the extension of the adsorption time, the adsorption rate at this stage decreases due to the gradual decrease in adsorption sites on the surface of the adsorbent. When the surface and internal pore adsorption saturate, the adsorption and desorption rates are comparable and the adsorption is in a dynamic equilibrium, leading to stabilization of the COD removal rate. As can be seen from Figure 8, the adsorption equilibrium time of BT and CTS–BT is approximately 100 min, while the adsorption equilibrium time of C18CTS−BT is shortened to 60 min, and the adsorption efficiency is greatly improved.

### 3.6. Performance of Adsorbent in Reducing COD of Coking Wastewater

Based on the above results, the optimal treatment process of C18CTS–BT for the coking wastewater is determined, i.e., the adsorbent dosage is 1.5 g·L^−1^, the adsorption time is 60 min, and the pH value of the coking wastewater is 7.2 without adjustment. Under these conditions, the performance of three different adsorbents for the COD reduction of coking wastewater is evaluated. The COD values of coking wastewater treated with BT, CTS–BT, and C18CTS–BT are reduced from 342 mg·L^−1^ to 264 mg·L^−1^, 218 mg·L^−1^, and 146 mg·L^−1^, corresponding to COD removal rates of 22.81%, 36.26%, and 57.31%, respectively. The above results show that the performance of C18CS–BT in reducing the COD of coking wastewater is further improved by loading bentonite after the hydrophobic modification of chitosan compared to the performance of CTS–BT.

Changes in organic composition in the coking wastewater before and after the treatment with C18CTS–BT were further analyzed by GC–MS. A total of 93 organic compounds were detected in the untreated raw wastewater, corresponding to 93 characteristic peaks in the total ion flow diagram in Figure S1a. For the coking wastewater treated with C18CTS–BT, we detected a total of 41 organic compounds, corresponding to 41 characteristic peaks in the total ion flow diagram in Figure S1b. In comparison, it was found that the reduction of organic species in the coking wastewater treated with C18CS–BT was about 55.91%. The results of GC–MS analysis of coking wastewater before and after the treatment with C18CTS–BT are shown in Table 1, taking the top ten major organic compounds in untreated coking wastewater in terms of organic content (characteristic peak integrated area) from high to low as a reference. From Table 1, it can be seen that the main characteristic organic compounds in the raw water of coking wastewater are unsaturated hydrocarbons, phenol and its derivatives, long–chain alkanes and their derivatives, etc. After the treatment with C18CTS–BT, unsaturated hydrocarbons, phenols, and their derivatives are the main characteristic organic compounds in coking wastewater, while long–chain alkanes and their derivatives were not detected.

## 4. Conclusions

In this work, we successfully prepared an amphiphilic composite adsorbent C18CTS–BT for the treatment of coking wastewater. The adsorption performance of C18CTS–BT in coking wastewater was further enhanced compared to those of raw BT and unhydrophobically modified CTS–BT. C18CTS–BT has the performance advantages of having a lower dosage, a wider pH application range, and a smaller adsorption equilibrium time, as well as the strongest capability to reduce the COD of coking wastewater. After the C18CTS–BT treatment, the types of dissolved organic compounds in coking wastewater were substantially reduced, while some long-chain alkanes and their derivatives were completely removed. This work will provide a theoretical reference for the application of adsorbent materials in coking wastewater.

## Figures and Tables

**Table 1:** GC–MS analysis results of coking wastewater before and after C18CTS–BT treatment.

No.	Organic Compound	Raw Wastewater		Treated	
		t/s	A/%	t/s	A/%
1	phenol	6.728	8.55	6.728	7.05
2	methyl phenol	9.228	2.33	9.228	1.54
3	1–decene	19.921	13.74	19.922	30.12
4	2,4–di–tert-butyl phenol	20.833	2.39	20.827	2.55
5	dibutyl phthalate	30.515	2.90	-	-
6	heptadecane	37.297	2.30	-	-
7	1–chloro-nonadecane	40.038	2.04	-	-
8	bis(2–ethylhexyl) phthalate	40.514	3.84	40.508	3.13
9	3–methylhexacosane	41.244	2.51	-	-
10	octadecane	43.679	2.00	-	-

t is the flow time; A is the percentage of the integrated area of the characteristic peak; - represents non-detected compounds.

## Data Availability

Not applicable.

## Supplementary Materials

The following are available online at https://www.mdpi.com/article/10.3390/polym15061588/s1, Figure S1: Total ion flow diagram of coking wastewater before and after C18CS−BT treatment.
