# Supplementary material for: Preparation of Amphiphilic Chitosan-Loaded Bentonite Adsorbent and Its Performance in Removing Organic Matter from Coking Wastewater
Source: Polymers (Basel). 2023 Mar 22;15(6):1588. doi: 10.3390/polym15061588 (PMC10055804; doi:10.3390/polym15061588)
Supplement: Supplementary file 1 [file polymers-15-01588-s001.zip › C18CS-BT treated coking wastewater.pdf]

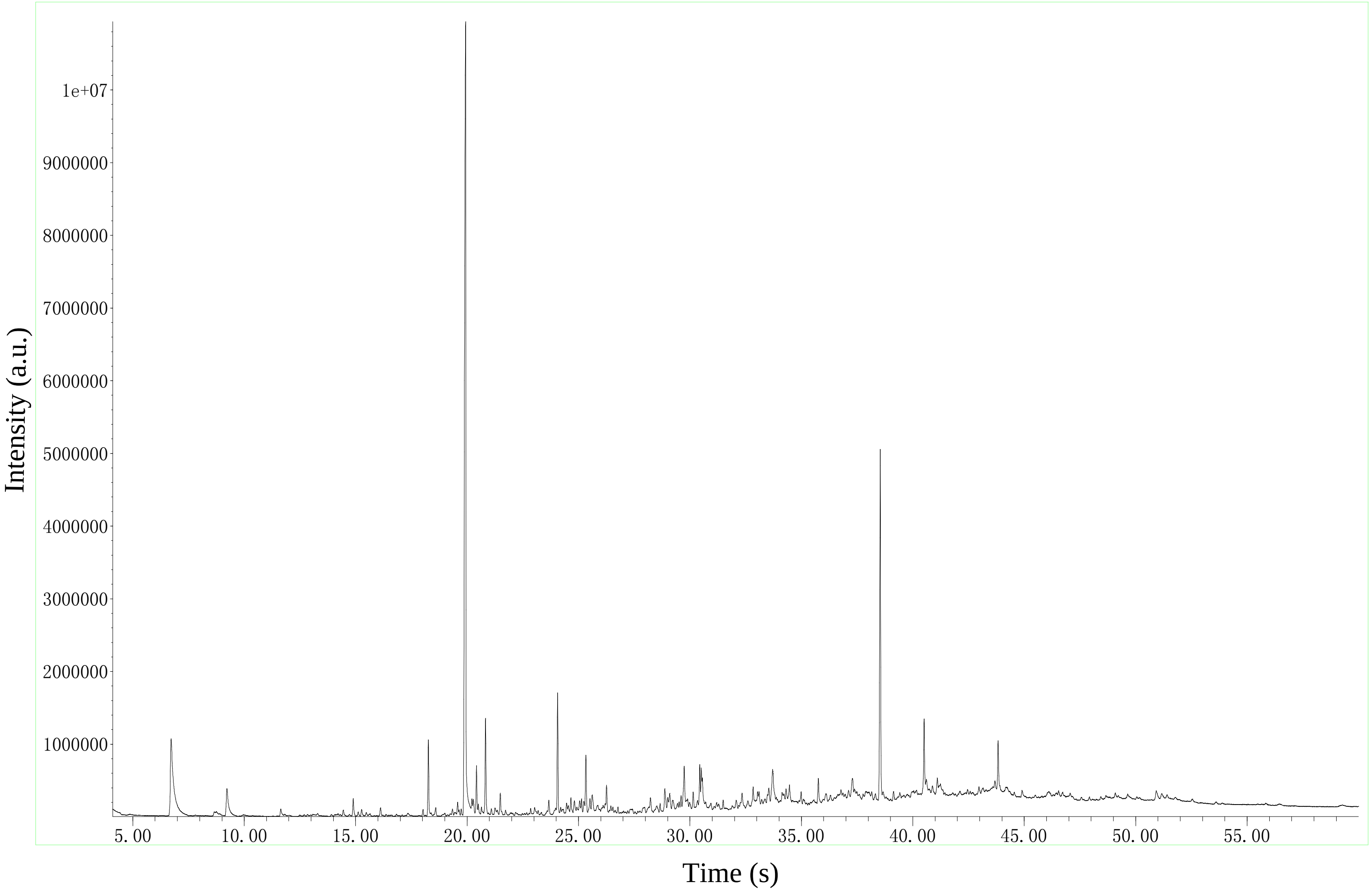

Intensity (a.u.)
1e+07
9000000
8000000
7000000
6000000
5000000
4000000
3000000
2000000
1000000
5.00
10.00
15.00
20.00
25.00
30.00
35.00
40.00
45.00
50.00
55.00
Time (s)

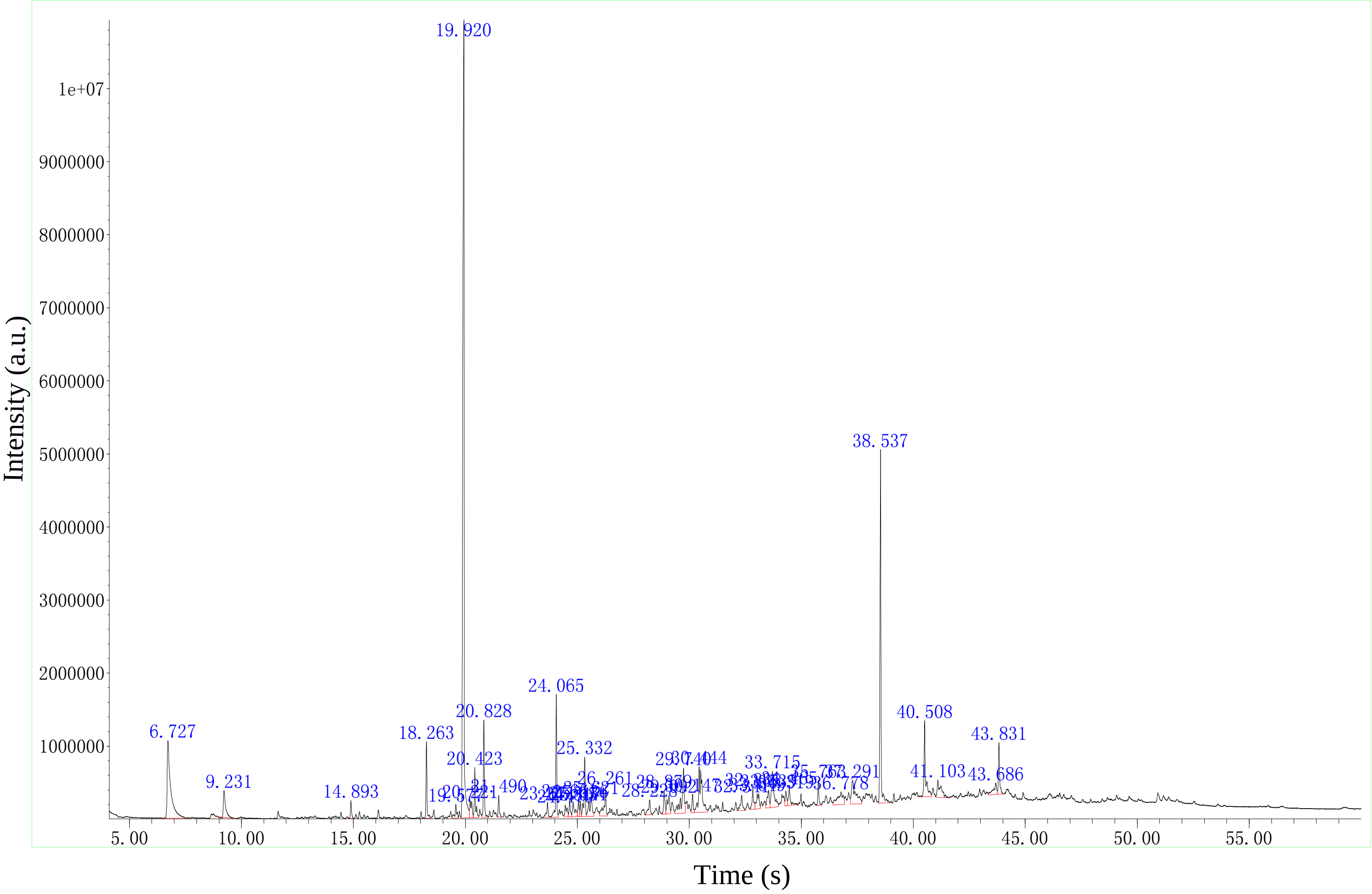

Intensity (a.u.)
Time (s)
1e+07
9000000
8000000
7000000
6000000
5000000
4000000
3000000
2000000
1000000
5.00
10.00
15.00
20.00
25.00
30.00
35.00
40.00
45.00
50.00
55.00
19.920
38.537
24.065
20.828
6.727
18.263
40.508
43.831
20.423
25.332
9.231
14.893
33.715
26.261
41.103
43.686
36.778

Spectrum Search Report

No.	R. T.	S%	Spectrum Library/ID	Reference#	CAS#	Match
1	6.728	7.05	C:\MassHunter\Library\NIST14.L			
			Phenol	2620	000108-95-2	94
			Phenol	2619	000108-95-2	91
			Phenol	2621	000108-95-2	91
2	9.228	1.54	C:\MassHunter\Library\NIST14.L			
			p-Cresol	5464	000106-44-5	97
			Phenol, 3-methyl-	5469	000108-39-4	96
			p-Cresol	5462	000106-44-5	94
3	14.892	0.51	C:\MassHunter\Library\NIST14.L			
			Sulfurous acid, hexyl pentyl ester	97941	1000309-14-1	58
			Dodecane, 2,6,11-trimethyl-	76621	031295-56-4	58
			Tetradecane, 1-iodo-	181794	019218-94-1	58
4	18.263	1.89	C:\MassHunter\Library\NIST14.L			
			Dodecanal	51252	000112-54-9	98
			Dodecanal	51251	000112-54-9	91
			Dodecanal	51253	000112-54-9	91
5	19.580	0.66	C:\MassHunter\Library\NIST14.L			
			Tridecane	51393	000629-50-5	93
			Hexadecane	89842	000544-76-3	91
			Hexadecane	89838	000544-76-3	86
6	19.922	30.12	C:\MassHunter\Library\NIST14.L			
			1-Decene	18385	000872-05-9	95
			1-Dodecanol	53008	000112-53-8	95
			1-Dodecanol	53012	000112-53-8	95
7	20.221	0.96	C:\MassHunter\Library\NIST14.L			
			Tetratetracontane	273586	007098-22-8	80
			Dodecane, 3-methyl-	51413	017312-57-1	64
			Hentriacontane	252712	000630-04-6	64
8	20.421	1.60	C:\MassHunter\Library\NIST14.L			
			Heneicosane	155888	000629-94-7	72
			Octadecane, 1-iodo-	226953	000629-93-6	72
			Decane, 2,3,7-trimethyl-	51447	062238-13-5	64
9	20.827	2.55	C:\MassHunter\Library\NIST14.L			
			2,4-Di-tert-butylphenol	70634	000096-76-4	97
			2,4-Di-tert-butylphenol	70633	000096-76-4	93
			2,4-Di-tert-butylphenol	70632	000096-76-4	92
10	21.492	0.63	C:\MassHunter\Library\NIST14.L			
			2-Bromo dodecane	109279	013187-99-0	90
			Hexadecane, 1-iodo-	206779	000544-77-4	80
			Heneicosane	155887	000629-94-7	72
11	23.668	0.63	C:\MassHunter\Library\NIST14.L			
			Heneicosane	155888	000629-94-7	86
			Hexadecane	89844	000544-76-3	86
			Triacontane	247876	000638-68-6	86
12	24.062	3.22	C:\MassHunter\Library\NIST14.L			
			Tributyl phosphate	126012	000126-73-8	91
			Tributyl phosphate	126016	000126-73-8	91
			Tributyl phosphate	126015	000126-73-8	86
13	24.468	0.66	C:\MassHunter\Library\NIST14.L			
			1,3,5-Triazine-2,4,6(1H,3H,5H)-tri	110268	001025-15-6	99

			one, 1,3,5-tri-2-propenyl-			
			1,3,5-Triazine-2,4,6(1H,3H,5H)-tri one, 1,3,5-tri-2-propenyl-	110269	001025-15-6	98
			1,3,5-Triazine-2,4,6(1H,3H,5H)-tri one, 1,3,5-tri-2-propenyl-	110270	001025-15-6	93
14	24.662	0.57	C:\MassHunter\Library\NIST14.L			
			Triacontane	247876	000638-68-6	86
			Heptadecane	102600	000629-78-7	80
			Dodecane, 2-methyl-	51414	001560-97-0	74
15	24.815	0.85	C:\MassHunter\Library\NIST14.L			
			Hexadecane	89842	000544-76-3	91
			Tridecane, 5-propyl-	89847	055045-11-9	80
			Tridecane, 3-methyl-	63633	006418-41-3	80
16	25.056	0.47	C:\MassHunter\Library\NIST14.L			
			Heneicosane	155888	000629-94-7	80
			2-Bromotetradecane	136205	074036-95-6	72
			Decane, 2,3,5-trimethyl-	51454	062238-11-3	72
17	25.133	0.44	C:\MassHunter\Library\NIST14.L			
			Hexadecane	89844	000544-76-3	91
			Hexadecane	89842	000544-76-3	87
			Pentadecane	76609	000629-62-9	83
18	25.333	1.98	C:\MassHunter\Library\NIST14.L			
			Heneicosane	155888	000629-94-7	72
			2-Bromotetradecane	136205	074036-95-6	72
			Tetratetracontane	273586	007098-22-8	64
19	25.621	1.49	C:\MassHunter\Library\NIST14.L			
			Dibenzylamine	62549	000103-49-1	91
			Dibenzylamine	62548	000103-49-1	68
			Dibenzylamine	62547	000103-49-1	64
20	26.262	1.53	C:\MassHunter\Library\NIST14.L			
			Pentacosane	207499	000629-99-2	86
			Hexadecane, 2-methyl-	102609	001560-92-5	59
			Dodecane, 4,6-dimethyl-	63643	061141-72-8	53
21	28.227	0.79	C:\MassHunter\Library\NIST14.L			
			Decane, 3,8-dimethyl-	40006	017312-55-9	87
			Octacosane	235614	000630-02-4	86
			Hexadecane, 2-methyl-	102609	001560-92-5	86
22	28.880	0.88	C:\MassHunter\Library\NIST14.L			
			Hexadecane, 1-chloro-	121206	004860-03-1	93
			Octadecane, 1-chloro-	148105	003386-33-2	90
			Hexadecane, 1-chloro-	121204	004860-03-1	89
23	29.092	1.15	C:\MassHunter\Library\NIST14.L			
			Heneicosane	155888	000629-94-7	91
			2-Bromotetradecane	136205	074036-95-6	86
			Octacosane	235614	000630-02-4	86
24	29.739	2.85	C:\MassHunter\Library\NIST14.L			
			Heneicosane	155888	000629-94-7	90
			Eicosane, 1-iodo-	241993	1000406-31-8	72
			Hexadecane, 2-methyl-	102609	001560-92-5	64
25	30.144	0.62	C:\MassHunter\Library\NIST14.L			

			Benzenepropanoic acid, 3,5-bis(1,1-dimethylethyl)-4-hydroxy-, methyl ester	151926	006386-38-5	99
			Benzenepropanoic acid, 3,5-bis(1,1-dimethylethyl)-4-hydroxy-, methyl ester	151925	006386-38-5	91
			Benzenepropanoic acid, 3,5-bis(1,1-dimethylethyl)-4-hydroxy-, methyl ester	151924	006386-38-5	89
26	30.444	4.12	C:\MassHunter\Library\NIST14.L			
			n-Hexadecanoic acid	117419	000057-10-3	97
			n-Hexadecanoic acid	117417	000057-10-3	89
			Ether, dodecyl isopropyl	91586	029379-42-8	58
27	32.338	0.82	C:\MassHunter\Library\NIST14.L			
			Heneicosane	155888	000629-94-7	93
			1,3-Propanediol, ethyl octadecyl ether	210760	1000406-35-4	90
			Nonadecane, 9-methyl-	142247	013287-24-6	89
28	32.838	0.91	C:\MassHunter\Library\NIST14.L			
			1-Octadecene	113633	000112-88-9	97
			1-Octadecene	113632	000112-88-9	96
			Pentadecafluorooctanoic acid, octadecyl ester	274630	1000406-04-8	93
29	33.050	1.09	C:\MassHunter\Library\NIST14.L			
			Hentriacontane	252711	000630-04-6	90
			Dotriacontane, 1-iodo-	272203	1000406-32-4	90
			Eicosane, 7-hexyl-	217896	055333-99-8	87
30	33.538	0.94	C:\MassHunter\Library\NIST14.L			
			Heptadecane, 9-octyl-	207503	007225-64-1	91
			Hentriacontane	252711	000630-04-6	91
			Pentacosane	207499	000629-99-2	91
31	33.715	2.52	C:\MassHunter\Library\NIST14.L			
			Octadecane	115547	000593-45-3	45
			Eicosane	142238	000112-95-8	43
			Tetracosane, 1-iodo-	260060	1000406-32-0	42
32	34.321	0.57	C:\MassHunter\Library\NIST14.L			
			Pentacosane	207499	000629-99-2	91
			Hentriacontane	252712	000630-04-6	90
			Docosane, 1-iodo-	252495	1000406-31-9	87
33	34.468	0.96	C:\MassHunter\Library\NIST14.L			
			Octadecane	115547	000593-45-3	93
			Heptacosane	227469	000593-49-7	90
			Octacosane	235614	000630-02-4	80
34	35.762	0.71	C:\MassHunter\Library\NIST14.L			
			Octanamide, N,N-dimethyl-	40697	001118-92-9	87
			N,N-Dimethyldodecanamide	90491	003007-53-2	72
			3-Cyclopentylpropionamide, N,N-dimethyl-	39063	1000340-38-0	64
35	36.779	1.75	C:\MassHunter\Library\NIST14.L			
			Hentriacontane	252712	000630-04-6	91
			Heptacosane	227468	000593-49-7	91
			Octacosane	235614	000630-02-4	90

36	37.291	2.61	C:\MassHunter\Library\NIST14.L			
			Hexadecane, 2-methyl-	102609	001560-92-5	90
			Pentacosane	207499	000629-99-2	76
			Hentriacontane	252711	000630-04-6	76
37	38.538	10.05	C:\MassHunter\Library\NIST14.L			
			Phenol, 2,2'-methylenebis[6-(1,1-dimethylethyl)-4-methyl-	197659	000119-47-1	93
			Phenol, 2,2'-methylenebis[6-(1,1-dimethylethyl)-4-methyl-	197662	000119-47-1	87
			Phenol, 2,2'-methylenebis[6-(1,1-dimethylethyl)-4-methyl-	197660	000119-47-1	74
38	40.508	3.13	C:\MassHunter\Library\NIST14.L			
			Bis(2-ethylhexyl) phthalate	233372	000117-81-7	91
			Bis(2-ethylhexyl) phthalate	233373	000117-81-7	87
			Phthalic acid, di(2-propylpentyl) ester	233419	1000377-93-5	87
39	41.102	1.60	C:\MassHunter\Library\NIST14.L			
			1,3-Propanediol, ethyl hexacosyl ether	261144	1000406-35-8	35
			Hexadecane, 2-methyl-	102608	001560-92-5	25
			1,3-Propanediol, docosyl ethyl ether	244133	1000406-35-6	20
40	43.685	0.94	C:\MassHunter\Library\NIST14.L			
			Octadecane, 1-iodo-	226951	000629-93-6	96
			Hexadecane, 2-methyl-	102609	001560-92-5	93
			Octadecane, 3-methyl-	128847	006561-44-0	93
41	43.832	1.65	C:\MassHunter\Library\NIST14.L			
			13-Docosenamide, (Z)-	194619	000112-84-5	94
			13-Docosenamide, (Z)-	194618	000112-84-5	90
			9-Octadecenamide, (Z)-	141028	000301-02-0	89
