# Supplementary material for: Preparation of Amphiphilic Chitosan-Loaded Bentonite Adsorbent and Its Performance in Removing Organic Matter from Coking Wastewater
Source: Polymers (Basel). 2023 Mar 22;15(6):1588. doi: 10.3390/polym15061588 (PMC10055804; doi:10.3390/polym15061588)
Supplement: Supplementary file 1 [file polymers-15-01588-s001.zip › Raw Wastewater.pdf]

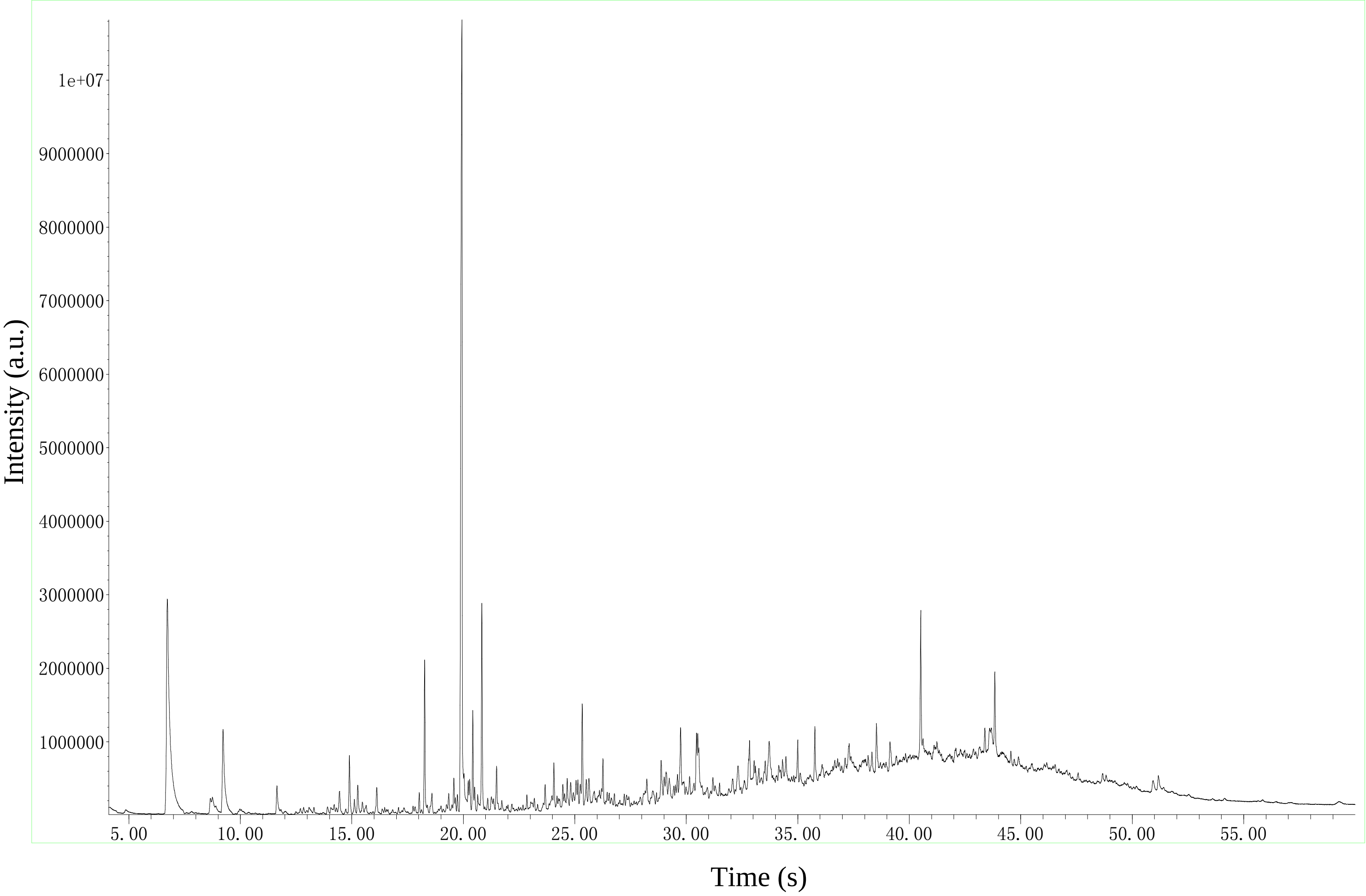

Intensity (a.u.)
1e+07
9000000
8000000
7000000
6000000
5000000
4000000
3000000
2000000
1000000
5.00
10.00
15.00
20.00
25.00
30.00
35.00
40.00
45.00
50.00
55.00
Time (s)

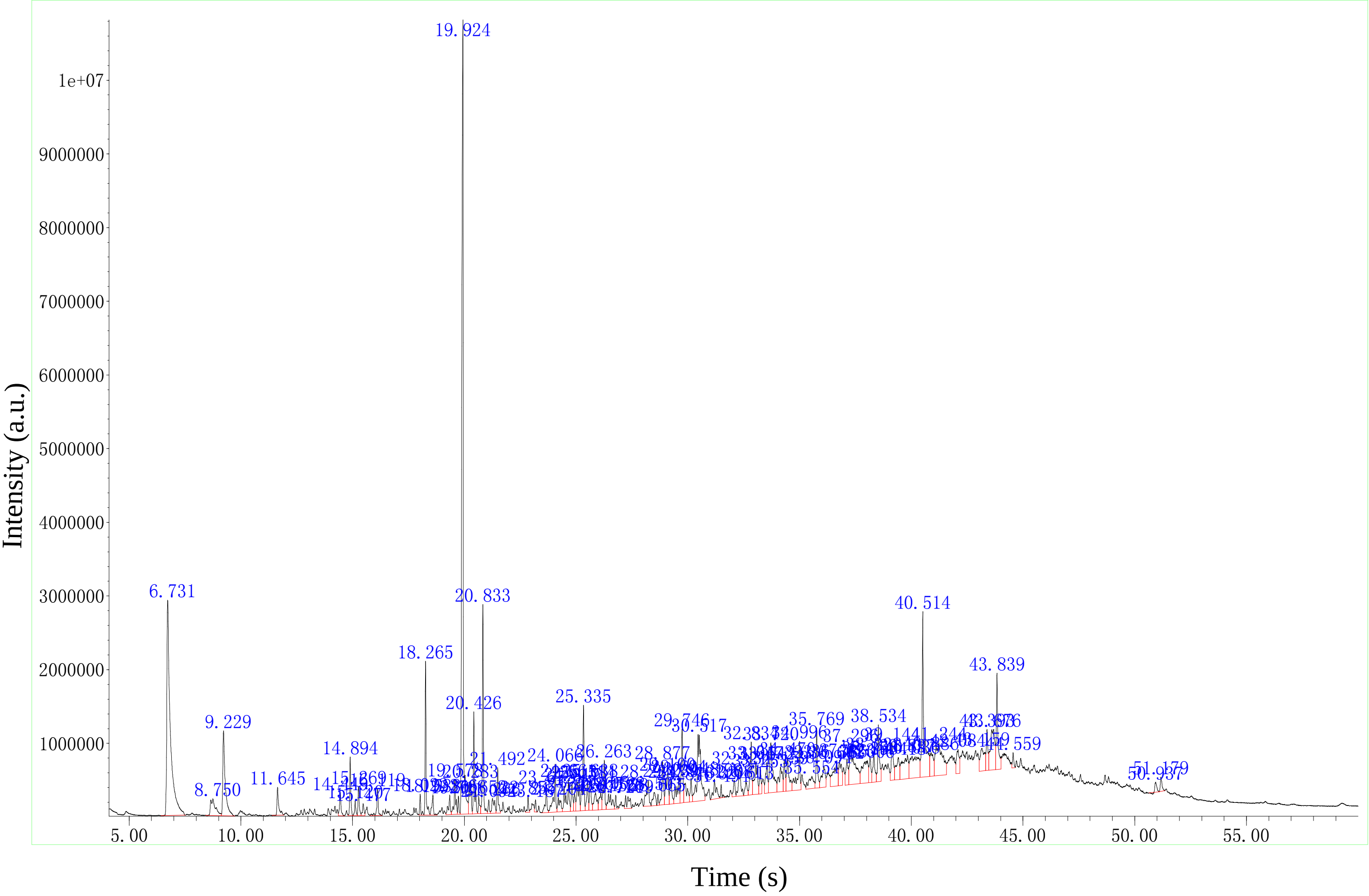

Intensity (a.u.)
Time (s)
1e+07
9000000
8000000
7000000
6000000
5000000
4000000
3000000
2000000
1000000
5.00
10.00
15.00
20.00
25.00
30.00
35.00
40.00
45.00
50.00
55.00
19.924
6.731
20.833
40.514
18.265
43.839
20.426
25.335
9.229
14.894
11.645
8.750
24.066
26.263
28.877
30.517
35.769
38.534

No.	R.T.	S%	Spectrum Library/ID	Reference#	CAS#	Match
1	6.728	8.55	C:\MassHunter\Library\NIST14.L			
			Phenol	2620	000108-95-2	94
			Phenol	2621	000108-95-2	91
			Phenol	2619	000108-95-2	91
2	8.752	0.91	C:\MassHunter\Library\NIST14.L			
			Phenol, 2-methyl-	5476	000095-48-7	94
			Phenol, 3-methyl-	5469	000108-39-4	83
			Phenol, 3-methyl-	5472	000108-39-4	52
3	9.228	2.33	C:\MassHunter\Library\NIST14.L			
			p-Cresol	5464	000106-44-5	97
			Phenol, 3-methyl-	5469	000108-39-4	96
			p-Cresol	5461	000106-44-5	96
4	11.646	0.53	C:\MassHunter\Library\NIST14.L			
			Silane, cyclohexyldimethoxymethyl-	55014	017865-32-6	87
			Silane, dimethoxydimethyl-	9350	001112-39-6	47
			Silane, dimethoxydimethyl-	9351	001112-39-6	40
5	14.451	0.38	C:\MassHunter\Library\NIST14.L			
			Octane, 2,4,6-trimethyl-	29378	062016-37-9	59
			Heptane, 2,4-dimethyl-	12960	002213-23-2	52
			Tetradecane, 1-iodo-	181794	019218-94-1	49
6	14.892	0.74	C:\MassHunter\Library\NIST14.L			
			Nonane, 5-methyl-5-propyl-	51475	017312-75-3	80
			Dodecane, 4,6-dimethyl-	63643	061141-72-8	72
			Octane, 5-ethyl-2-methyl-	29382	062016-18-6	72
7	15.122	0.21	C:\MassHunter\Library\NIST14.L			
			Dodecane	39973	000112-40-3	87
			Tetradecane	63625	000629-59-4	86
			Tridecane, 1-iodo-	168440	035599-77-0	80
8	15.269	0.41	C:\MassHunter\Library\NIST14.L			
			Indole	8731	000120-72-9	91
			Indole	8734	000120-72-9	90
			Benzyl nitrile	8743	000140-29-4	60
9	15.475	0.20	C:\MassHunter\Library\NIST14.L			
			Octane, 2-methyl-	12950	003221-61-2	64
			Dodecane, 1-iodo-	154845	004292-19-7	59
			Tridecane, 1-iodo-	168440	035599-77-0	59
10	16.116	0.45	C:\MassHunter\Library\NIST14.L			
			Dodecane	39972	000112-40-3	91
			Decane, 3,7-dimethyl-	39995	017312-54-8	72
			Sulfurous acid, hexyl pentyl ester	97941	1000309-14-1	64
11	18.022	0.26	C:\MassHunter\Library\NIST14.L			
			Tetradecane	63622	000629-59-4	98
			Tetradecane	63623	000629-59-4	96
			Tetradecane	63625	000629-59-4	96
12	18.263	1.69	C:\MassHunter\Library\NIST14.L			
			Tetradecanal	76509	000124-25-4	98
			Dodecanal	51252	000112-54-9	98
			Dodecanal	51251	000112-54-9	91
13	18.586	0.36	C:\MassHunter\Library\NIST14.L			
			Heneicosane	155888	000629-94-7	86

			Nonadecane, 9-methyl-	142247	013287-24-6	86
			Hexacosane	217890	000630-01-3	86
14	19.345	0.39	C:\MassHunter\Library\NIST14.L			
			2-methyloctacosane	242320	1000376-72-8	80
			Octadecane, 1-iodo-	226952	000629-93-6	74
			Decane, 3,8-dimethyl-	40006	017312-55-9	74
15	19.580	0.66	C:\MassHunter\Library\NIST14.L			
			Hexadecane	89842	000544-76-3	91
			Hentriacontane	252712	000630-04-6	86
			Decane, 2,3,6-trimethyl-	51446	062238-12-4	80
16	19.739	0.20	C:\MassHunter\Library\NIST14.L			
			Tetradecane, 1-iodo-	181793	019218-94-1	72
			Decane, 3,8-dimethyl-	40006	017312-55-9	64
			Nonadecane, 9-methyl-	142247	013287-24-6	58
17	19.921	13.74	C:\MassHunter\Library\NIST14.L			
			1-Decene	18385	000872-05-9	95
			1-Dodecanol	53012	000112-53-8	95
			1-Dodecanol	53008	000112-53-8	95
18	20.286	0.78	C:\MassHunter\Library\NIST14.L			
			Hexadecane	89842	000544-76-3	86
			Pentadecane	76609	000629-62-9	83
			Heptadecane	102598	000629-78-7	83
19	20.427	1.36	C:\MassHunter\Library\NIST14.L			
			Dodecane, 2,6,11-trimethyl-	76619	031295-56-4	59
			Heptadecane	102600	000629-78-7	59
			Sulfurous acid, 2-ethylhexyl isohexyl ester	137919	1000309-19-0	58
20	20.651	0.27	C:\MassHunter\Library\NIST14.L			
			1-Iodo-2-methylundecane	154846	073105-67-6	72
			Octadecane, 1-iodo-	226953	000629-93-6	72
			Octane, 2,4,6-trimethyl-	29378	062016-37-9	64
21	20.833	2.39	C:\MassHunter\Library\NIST14.L			
			2,4-Di-tert-butylphenol	70632	000096-76-4	96
			Phenol, 2,5-bis(1,1-dimethylethyl)	70651	005875-45-6	95
			2,4-Di-tert-butylphenol	70634	000096-76-4	95
22	21.092	0.18	C:\MassHunter\Library\NIST14.L			
			Sulfurous acid, pentadecyl 2-propyl ester	191792	1000309-12-6	72
			Sulfurous acid, 2-propyl tridecyl ester	164999	1000309-12-4	64
			Disulfide, di-tert-dodecyl	239746	027458-90-8	64
23	21.268	0.45	C:\MassHunter\Library\NIST14.L			
			Tetratetracontane	273586	007098-22-8	87
			Hentriacontane	252712	000630-04-6	87
			Heptacosane	227468	000593-49-7	86
24	21.492	0.62	C:\MassHunter\Library\NIST14.L			
			Heneicosane	155888	000629-94-7	80
			Dodecane, 1-iodo-	154845	004292-19-7	64
			Hexane, 2,3,4-trimethyl-	12990	000921-47-1	58
25	22.857	0.20	C:\MassHunter\Library\NIST14.L			

			Hexadecane	89843	000544-76-3	94
			Hexadecane	89840	000544-76-3	92
			Hexadecane	89838	000544-76-3	92
26	23.186	0.14	C:\MassHunter\Library\NIST14.L			
			Tridecanol, 2-ethyl-2-methyl-	104449	1000115-66-1	52
			Pentacosane	207499	000629-99-2	52
			Heneicosane	155888	000629-94-7	52
27	23.674	0.47	C:\MassHunter\Library\NIST14.L			
			Heneicosane	155888	000629-94-7	87
			2-Bromo dodecane	109279	013187-99-0	87
			Hexadecane	89844	000544-76-3	86
28	24.068	0.98	C:\MassHunter\Library\NIST14.L			
			Tributyl phosphate	126012	000126-73-8	83
			Tributyl phosphate	126014	000126-73-8	80
			Tributyl phosphate	126016	000126-73-8	80
29	24.204	0.44	C:\MassHunter\Library\NIST14.L			
			Dodecane, 2-methyl-	51414	001560-97-0	91
			Decane, 2,3,7-trimethyl-	51447	062238-13-5	86
			Nonadecane	128834	000629-92-5	83
30	24.662	1.02	C:\MassHunter\Library\NIST14.L			
			Heneicosane	155887	000629-94-7	90
			Heneicosane	155888	000629-94-7	87
			Tetracosane	195669	000646-31-1	86
31	24.815	0.47	C:\MassHunter\Library\NIST14.L			
			Nonadecane	128835	000629-92-5	90
			Hexadecane	89844	000544-76-3	80
			Heneicosane	155888	000629-94-7	80
32	24.939	0.28	C:\MassHunter\Library\NIST14.L			
			Hexacosane	217890	000630-01-3	87
			Octacosane	235614	000630-02-4	81
			Triacontane	247876	000638-68-6	72
33	25.139	0.81	C:\MassHunter\Library\NIST14.L			
			Dodecane, 2-methyl-	51414	001560-97-0	83
			Heneicosane	155888	000629-94-7	80
			Octadecane, 1-iodo-	226953	000629-93-6	80
34	25.333	1.51	C:\MassHunter\Library\NIST14.L			
			Heneicosane	155888	000629-94-7	90
			Heptadecane, 2,6,10,15-tetramethyl	155904	054833-48-6	86
			Hexadecane, 1-iodo-	206779	000544-77-4	72
35	25.515	0.51	C:\MassHunter\Library\NIST14.L			
			2-Bromotetradecane	136205	074036-95-6	86
			Heptadecane	102600	000629-78-7	80
			Heneicosane	155888	000629-94-7	80
36	25.639	0.55	C:\MassHunter\Library\NIST14.L			
			Dotriacontane, 1-iodo-	272203	1000406-32-4	46
			Decane, 3,8-dimethyl-	40006	017312-55-9	46
			Hentriacontane	252712	000630-04-6	46
37	25.868	0.58	C:\MassHunter\Library\NIST14.L			
			Tridecanol, 2-ethyl-2-methyl-	104449	1000115-66-1	80
			Heneicosane	155888	000629-94-7	80

			Hexacosane	217891	000630-01-3	80
38	26.103	0.44	C:\MassHunter\Library\NIST14.L			
			Heneicosane	155888	000629-94-7	87
			Hexacosane	217890	000630-01-3	87
			Heptacosane	227468	000593-49-7	83
39	26.262	0.81	C:\MassHunter\Library\NIST14.L			
			1,3-Propanediol, ethyl hexadecyl ether	186677	1000406-35-3	86
			Heneicosane	155888	000629-94-7	86
			Pentacosane	207499	000629-99-2	86
40	26.456	0.65	C:\MassHunter\Library\NIST14.L			
			Dodecane, 2-methyl-	51414	001560-97-0	87
			Octacosane	235614	000630-02-4	87
			Nonane, 5-butyl-	51397	017312-63-9	86
41	26.774	0.21	C:\MassHunter\Library\NIST14.L			
			Octadecane	115545	000593-45-3	64
			Heneicosane	155888	000629-94-7	64
			2-Bromotetradecane	136205	074036-95-6	64
42	27.221	0.48	C:\MassHunter\Library\NIST14.L			
			Octadecane	115546	000593-45-3	96
			Octadecane	115547	000593-45-3	96
			Octadecane	115545	000593-45-3	93
43	28.233	0.73	C:\MassHunter\Library\NIST14.L			
			Heneicosane	155888	000629-94-7	90
			Hexadecane	89844	000544-76-3	83
			2-methyloctacosane	242320	1000376-72-8	80
44	28.503	0.41	C:\MassHunter\Library\NIST14.L			
			Octacosane	235614	000630-02-4	91
			Hentriacontane	252711	000630-04-6	91
			Hexacosane	217890	000630-01-3	91
45	28.668	0.12	C:\MassHunter\Library\NIST14.L			
			Phthalic acid, butyl tetradecyl ester	246447	1000308-91-3	80
			Phthalic acid, butyl undecyl ester	224935	1000308-91-2	80
			Phthalic acid, 2,4-dimethylpent-3-yl isobutyl ester	178903	1000356-84-3	72
46	28.880	0.80	C:\MassHunter\Library\NIST14.L			
			Hexadecane, 1-chloro-	121204	004860-03-1	96
			Octadecane, 1-chloro-	148105	003386-33-2	91
			Tritetracontane	273205	007098-21-7	91
47	29.097	0.94	C:\MassHunter\Library\NIST14.L			
			Eicosane	142238	000112-95-8	91
			Octacosane	235614	000630-02-4	91
			Pentadecane, 2,6,10-trimethyl-	115565	003892-00-0	90
48	29.238	0.57	C:\MassHunter\Library\NIST14.L			
			Heptadecane	102599	000629-78-7	94
			Heneicosane	155888	000629-94-7	93
			Eicosane	142238	000112-95-8	91
49	29.603	0.81	C:\MassHunter\Library\NIST14.L			
			Pentacosane	207499	000629-99-2	91

			Eicosane, 2-methyl-	155892	001560-84-5	90
			Octadecane, 1-iodo-	226951	000629-93-6	87
50	29.744	1.29	C:\MassHunter\Library\NIST14.L			
			Octacosane	235614	000630-02-4	90
			Octadecane, 1-iodo-	226953	000629-93-6	90
			Eicosane	142238	000112-95-8	72
51	29.874	0.69	C:\MassHunter\Library\NIST14.L			
			Octacosane	235614	000630-02-4	87
			Hexacosane	217891	000630-01-3	87
			Eicosane, 1-iodo-	241993	1000406-31-8	87
52	30.150	0.39	C:\MassHunter\Library\NIST14.L			
			Benzenepropanoic acid, 3,5-bis(1,1-dimethylethyl)-4-hydroxy-, methyl ester	151926	006386-38-5	97
			Benzenepropanoic acid, 3,5-bis(1,1-dimethylethyl)-4-hydroxy-, methyl ester	151925	006386-38-5	91
			Benzenepropanoic acid, 3,5-bis(1,1-dimethylethyl)-4-hydroxy-, methyl ester	151924	006386-38-5	78
53	30.515	2.90	C:\MassHunter\Library\NIST14.L			
			Dibutyl phthalate	138057	000084-74-2	95
			Dibutyl phthalate	138058	000084-74-2	94
			1,2-Benzenedicarboxylic acid, butyl octyl ester	192151	000084-78-6	86
54	31.197	0.63	C:\MassHunter\Library\NIST14.L			
			Eicosane	142239	000112-95-8	89
			Octadecane, 1-chloro-	148105	003386-33-2	64
			Hexacosane	217890	000630-01-3	50
55	31.491	0.17	C:\MassHunter\Library\NIST14.L			
			2-Bromo dodecane	109279	013187-99-0	70
			Dotriacontane, 1-iodo-	272203	1000406-32-4	68
			Heneicosane	155886	000629-94-7	68
56	32.079	0.32	C:\MassHunter\Library\NIST14.L			
			Benzo[d]pyrazolo[3,4-b]azepin-3(2H)-one, 5,6-dihydro-5,5-dimethyl-	90344	263550-89-6	70
			Dicyclopenta[a,d]benzene, 4,8-diethyl-1,5-dimethyl-	104478	1000156-41-6	70
			5-Methoxy-2-methyl-4-oxo-1,2,3,4-tetrahydro-1,10-phenantthroline	104038	1000227-06-0	49
57	32.321	0.78	C:\MassHunter\Library\NIST14.L			
			Fluoranthene	66901	000206-44-0	83
			Hexadecane, 2,6,10,14-tetramethyl-	142261	000638-36-8	64
			Benzene, 1,1'-(1,3-butadiyne-1,4-diyl)bis-	66906	000886-66-8	52
58	32.615	0.33	C:\MassHunter\Library\NIST14.L			
			Hexadecane, 2-methyl-	102609	001560-92-5	89
			Dotriacontane, 1-iodo-	272203	1000406-32-4	87
			2-methyloctacosane	242320	1000376-72-8	83
59	32.832	1.01	C:\MassHunter\Library\NIST14.L			
			1-Octadecene	113633	000112-88-9	97
			Z-8-Hexadecene	87835	1000130-87-5	95

			Pentadecafluorooctanoic acid, octadecyl ester	274630	1000406-04-8	93
60	33.050	1.12	C:\MassHunter\Library\NIST14.L			
			Heneicosane	155886	000629-94-7	97
			Hexadecane, 2,6,10,14-tetramethyl-	142261	000638-36-8	91
			Nonadecane, 9-methyl-	142247	013287-24-6	90
61	33.250	0.44	C:\MassHunter\Library\NIST14.L			
			Pyrene	66899	000129-00-0	91
			Pyrene	66896	000129-00-0	83
			Pyrene	66897	000129-00-0	64
62	33.544	0.73	C:\MassHunter\Library\NIST14.L			
			Hentriacontane	252711	000630-04-6	91
			Octacosane	235614	000630-02-4	91
			Dotriacontane, 1-iodo-	272203	1000406-32-4	90
63	33.721	1.86	C:\MassHunter\Library\NIST14.L			
			Hentriacontane	252711	000630-04-6	70
			Pentacosane	207499	000629-99-2	70
			Eicosane	142238	000112-95-8	62
64	34.162	0.97	C:\MassHunter\Library\NIST14.L			
			Heptadecane, 3-methyl-	115552	006418-44-6	83
			Nonadecane, 1-chloro-	161769	062016-76-6	83
			1-Bromodocosane	232142	006938-66-5	74
65	34.321	0.53	C:\MassHunter\Library\NIST14.L			
			Octacosane	235614	000630-02-4	91
			Hentriacontane	252711	000630-04-6	91
			2-methyloctacosane	242320	1000376-72-8	90
66	34.468	0.96	C:\MassHunter\Library\NIST14.L			
			Octacosane	235614	000630-02-4	91
			Octadecane	115547	000593-45-3	86
			Hentriacontane	252711	000630-04-6	86
67	34.997	1.24	C:\MassHunter\Library\NIST14.L			
			Cycloeicosane	140274	000296-56-0	95
			1-Octadecene	113633	000112-88-9	91
			2- Chloropropionic acid, hexadecyl ester	190088	086711-81-1	91
68	35.556	0.68	C:\MassHunter\Library\NIST14.L			
			1-Chloroeicosane	175300	042217-02-7	93
			Hexadecane, 2,6,10,14-tetramethyl-	142260	000638-36-8	83
			Carbonic acid, eicosyl vinyl ester	219345	1000382-54-3	76
69	35.767	0.89	C:\MassHunter\Library\NIST14.L			
			Octanamide, N,N-dimethyl-	40697	001118-92-9	87
			N,N-Dimethyldodecanamide	90491	003007-53-2	72
			3-Cyclopentylpropionamide, N,N-dimethyl-	39063	1000340-38-0	64
70	36.097	0.83	C:\MassHunter\Library\NIST14.L			
			Carbonic acid, eicosyl vinyl ester	219345	1000382-54-3	76
			Sulfurous acid, butyl heptadecyl ester	224827	1000309-18-4	74
			Nonadecane, 1-chloro-	161769	062016-76-6	70
71	36.662	1.23	C:\MassHunter\Library\NIST14.L			

			Octadecane, 1-chloro-	148105	003386-33-2	78
			Nonadecane, 1-chloro-	161769	062016-76-6	78
			Octadecane, 3-methyl-	128847	006561-44-0	62
72	36.785	0.78	C:\MassHunter\Library\NIST14.L			
			Docosane	169409	000629-97-0	96
			Heptadecane, 3-methyl-	115552	006418-44-6	81
			Dotriacontane, 1-iodo-	272203	1000406-32-4	81
73	37.120	0.63	C:\MassHunter\Library\NIST14.L			
			2-Methyltetracosane	207500	001560-78-7	87
			Hentriacontane	252711	000630-04-6	83
			Tetracosane, 11-decyl-	262990	055429-84-0	83
74	37.297	2.30	C:\MassHunter\Library\NIST14.L			
			Heptadecane	102600	000629-78-7	91
			Hexadecane, 2,6,10,14-tetramethyl-	142260	000638-36-8	89
			Tetracosane, 9-octyl-	256759	055401-54-2	87
75	38.009	1.50	C:\MassHunter\Library\NIST14.L			
			Hexadecane	89844	000544-76-3	92
			Hexadecane	89838	000544-76-3	91
			Hexadecane	89842	000544-76-3	91
76	38.156	0.53	C:\MassHunter\Library\NIST14.L			
			Heneicosane	155886	000629-94-7	93
			Heptadecane, 3-methyl-	115552	006418-44-6	89
			Nonadecane, 9-methyl-	142247	013287-24-6	78
77	38.326	0.67	C:\MassHunter\Library\NIST14.L			
			Hexadecane, 1-iodo-	206777	000544-77-4	91
			Henicos-1-ene	153971	001599-68-4	89
			Carbonic acid, eicosyl vinyl ester	219345	1000382-54-3	87
78	38.532	1.10	C:\MassHunter\Library\NIST14.L			
			Phenol, 2,2'-methylenebis[6-(1,1-dimethylethyl)-4-methyl-	197659	000119-47-1	99
			Phenol, 2,2'-methylenebis[6-(1,1-dimethylethyl)-4-methyl-	197660	000119-47-1	70
			Phenol, 2,2'-methylenebis[6-(1,1-dimethylethyl)-4-methyl-	197661	000119-47-1	68
79	39.144	0.88	C:\MassHunter\Library\NIST14.L			
			N,N-Dimethyldodecanamide	90491	003007-53-2	55
			3-Cyclopentylpropionamide, N,N-dimethyl-	39063	1000340-38-0	50
			2-Tridecanone, O-methyloxime	90497	036379-38-1	43
80	39.420	0.86	C:\MassHunter\Library\NIST14.L			
			Hexadecane	89839	000544-76-3	95
			1-Chloroeicosane	175300	042217-02-7	93
			Nonahexacontanoic acid	276185	040710-32-5	91
81	39.838	1.56	C:\MassHunter\Library\NIST14.L			
			Nonadecane	128833	000629-92-5	91
			Docosane	169409	000629-97-0	90
			Hexadecane, 1-iodo-	206777	000544-77-4	90
82	40.038	2.04	C:\MassHunter\Library\NIST14.L			
			Nonadecane, 1-chloro-	161769	062016-76-6	83
			Carbonic acid, eicosyl vinyl ester	219345	1000382-54-3	81
			Tetracosane	195672	000646-31-1	76

83	40.514	3.84	C:\MassHunter\Library\NIST14.L			
			Bis(2-ethylhexyl) phthalate	233372	000117-81-7	99
			Di-n-octyl phthalate	233365	000117-84-0	91
			Diisooctyl phthalate	233366	000131-20-4	91
84	40.885	0.90	C:\MassHunter\Library\NIST14.L			
			Heneicosane	155886	000629-94-7	97
			Hexadecane, 1-iodo-	206777	000544-77-4	92
			Octadecane, 2,6,10,14-tetramethyl-	169419	054964-82-8	91
85	41.244	2.51	C:\MassHunter\Library\NIST14.L			
			3-Methylhexacosane	227471	065820-56-6	91
			2-methyloctacosane	242320	1000376-72-8	87
			Dotriacontane, 1-iodo-	272203	1000406-32-4	87
86	42.085	0.70	C:\MassHunter\Library\NIST14.L			
			1-Bromodocosane	232142	006938-66-5	92
			Hexadecane, 1-iodo-	206777	000544-77-4	92
			Heneicosane	155886	000629-94-7	92
87	43.161	1.14	C:\MassHunter\Library\NIST14.L			
			Docosane	169409	000629-97-0	95
			Octadecane, 3-methyl-	128847	006561-44-0	93
			13-Methylheptacosane	235622	015689-72-2	90
88	43.391	0.76	C:\MassHunter\Library\NIST14.L			
			1,4-Benzenedicarboxylic acid, bis(2-ethylhexyl) ester	233538	006422-86-2	43
			Carbonic acid, decyl 2-ethylhexyl ester	173326	1000383-13-7	38
			Carbonic acid, 2-ethylhexyl nonyl ester	159890	1000383-13-6	38
89	43.679	2.00	C:\MassHunter\Library\NIST14.L			
			Octadecane	115545	000593-45-3	91
			2,6,10-Trimethyltridecane	89861	003891-99-4	80
			Heneicosane, 11-decyl-	252715	055320-06-4	76
90	43.838	1.70	C:\MassHunter\Library\NIST14.L			
			13-Docosenamide, (Z)-	194617	000112-84-5	97
			13-Docosenamide, (Z)-	194619	000112-84-5	92
			13-Docosenamide, (Z)-	194618	000112-84-5	83
91	44.561	0.21	C:\MassHunter\Library\NIST14.L			
			Heptacosane, 1-chloro-	245034	062016-79-9	96
			Heneicosane	155886	000629-94-7	89
			Nonadecane	128835	000629-92-5	86
92	50.937	0.19	C:\MassHunter\Library\NIST14.L			
			Di-n-decylsulfone	202597	111530-37-1	53
			1H-Indole, 5-methyl-2-phenyl-	71661	013228-36-9	49
			Heneicosane, 3-methyl-	169412	006418-47-9	45
93	51.178	0.27	C:\MassHunter\Library\NIST14.L			
			Eicosane	142239	000112-95-8	60
			Heptadecane	102600	000629-78-7	60
			Heneicosane	155886	000629-94-7	53
