# Supplementary material for: Preparation of Amphiphilic Chitosan-Loaded Bentonite Adsorbent and Its Performance in Removing Organic Matter from Coking Wastewater
Source: Polymers (Basel). 2023 Mar 22;15(6):1588. doi: 10.3390/polym15061588 (PMC10055804; doi:10.3390/polym15061588)
Supplement: Supplementary file 1 [file polymers-15-01588-s001.zip › supplementary material.pdf]

Preparation of Amphiphilic Chitosan-loaded Bentonite Adsorbent and Its Performance in Removing Organic Matter from Coking Wastewater

Zhou Zhu [1, 2, *], Haiqun Kou [1, 2], Yuchang Zhou [1], Xindian Lan [1], Meiying Yu [1], Haonan Chen [1]

[1] School of Ecology and Environment, Yuzhang Normal University, Nanchang 330103, P. R. China

[2] Key Laboratory of Green New Materials and Industrial Wastewater Treatment of Nanchang City, Yuzhang Normal University, Nanchang 330103, P. R. China

Corresponding author e-mail: zhuzhou20000@126.com

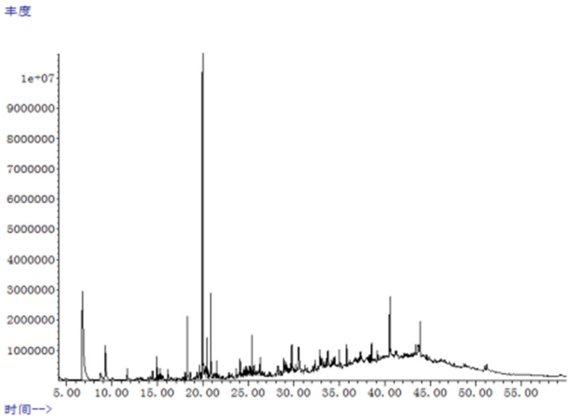

(a) Raw Wastewater

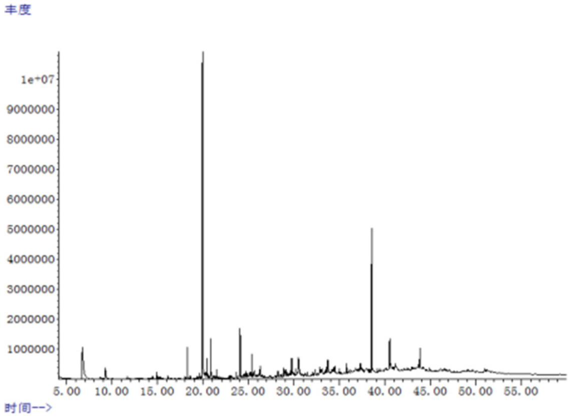

(b) C18CS-BT treated coking wastewater

Figure S1. Total ion flow diagram of coking wastewater before and after C18CS-BT treatment.
